# Simple and smart—promoting consumers’ willingness to consume and offer expired but still edible food through an informational intervention

**DOI:** 10.3389/fpsyg.2025.1514312

**Published:** 2025-09-12

**Authors:** Karolin Schmidt

**Affiliations:** Faculty of Natural Sciences, Institute of Psychology, Environmental Psychology, Otto-von-Guericke-University Magdeburg, Magdeburg, Germany

**Keywords:** food waste, intervention practice, consumer behavior, informational intervention, date labels

## Abstract

**Introduction:**

In order to curb household food waste in industrialized countries such as Germany, appropriate interventions are needed to encourage consumers to adopt various food-waste-prevention practices, for example, with respect to expired food. The main objective of the present study was to evaluate the effects of an informational intervention. This intervention provided problem and action knowledge about the environmental problem of household food waste and consumers’ engagement in food-waste-prevention consumption practices referring to expired food. The study focused on consumers’ willingness to consume and to offer expired but still edible food. Additionally, it examined the psychological mechanisms underlying these effects.

**Methods:**

We conducted an online survey in a sample of German consumers (*N* = 558). For the survey, participants were randomly assigned to an experimental group (EG, which was given the informational intervention) or a control group (CG, which was given a placebo intervention).

**Result and discussion:**

In line with our expectations, we found that EG participants reported a stronger personal norm for the consumption of expired but still edible food as well as lower perceived health risks when consuming expired food than CG participants did. Furthermore, EG participants were significantly more willing to offer expired but still edible food to others in a hypothetical food-choice experiment than CG participants were. A mediation analysis implied this intervention effect to be mediated by participants’ personal norm and their perceived health risks. Taken together, the present study provides valuable insights for an intervention designed to prevent household food waste by focusing on relevant consumption practices and going beyond a consumer-focused intervention perspective.

## 1 Introduction

After the United Nations Food Waste Index Report was issued in 2022, 1.05 billion tons of food were wasted (i.e., discarded, not consumed in a timely manner, or deemed unsuitable for human consumption; see, e.g., Thyberg and Tonjes, 2016) in the retail, food service, and household sectors combined. Out of the 132 kg of food waste that resulted per capita per year, 79 kg of food waste per capita per year came from households (Forbes et al., 2024). In 2015, the United Nations committed to the *Sustainable Development Goal* (SDG) 12.3, which prescribed the target to cut per capita global food waste at the retail and consumer levels in half […] by 2030 (United Nations, 2024). However, achieving this goal still seems a long way off, and hence, effective ways to prevent household food waste are still needed.

In this context, research has provided comprehensive empirical evidence for the high relevance of consumers’ various daily consumption practices for the prevention of household food waste (see, e.g., Roodhuyzen et al., 2017; Schanes et al., 2018; Schmidt and Matthies, 2018). In addition to consumption practices that refer to grocery shopping, food storage, and meal preparation, consumers’ engagement in *food-*waste-prevention consumption practices *with respect to expired food* has been shown to be highly relevant for effectively preventing household food waste (Aschemann-Witzel et al., 2015; Buzby et al., 2014; Schmidt and Matthies, 2018). Thus, consumption practices such as avoiding the immediate disposal of expired food, performing further sensory checks (e.g., smelling or tasting to assess edibility), and ultimately consuming expired but still edible food are considered effective strategies for reducing household food waste. In this context, the term “expired food” refers to the fact that foodstuff has deviated from normal/optimal foods, for example, with regard to relevant date labels. Thereby, most food products, which are placed on the market are labeled with either the minimum durability (presented by the “best-before” date label) or “use-by” date labels. Although various definitions of date labels exist in the research literature, the present paper adopts the definition that the best-before date indicates the point after which a food product may no longer meet expected quality standards, but is still considered safe to consume. In contrast, the use-by date gives the information that after the mentioned date the food product should not be consumed – even if it looks, smells and tastes good (see, e.g., Dordevic et al., 2020 for an overview; European Parliament and of the Council, 2011). Since it is difficult to make general statements about how long a food product remains edible – and therefore completely safe to consume – after the best-before date has passed (as factors such as proper storage significantly influence a food’s shelf life and edibility), further sensory testing is always a suitable strategy for assessing the edibility of products past their best-before date. Nevertheless, many food products – if stored correctly – are usually still considered edible and safe to eat a few days or even weeks after the best-before date expired. For example, with regard to dairy products, cheese and yoghurt (unopened) are considered to be edible for at least several days or weeks (see, e.g., Verbraucherzentrale Hamburg, 2019 for examples). Against this background, in the present paper, the term “expired but still edible food” exclusively refers to food products being close to or beyond the best-before date, but not being close to or beyond a use-by date, thus, the term refers to food products, which are still safe to eat.

Date labels on food products generally play a crucial role in communication between food manufacturers and consumers. They serve as an important basis for consumers to make informed purchasing decisions. However, many consumers report significant confusion regarding the meanings and implications of different date labels. Numerous studies suggest that this confusion – particularly between best-before and use-by dates – is a key factor contributing to household food waste (e.g., best-before vs. use-by dates; see, e.g., Patra et al., 2022; Gong et al., 2022; Shamim et al., 2022; Kavanaugh and Quinlan, 2020; Priefer et al., 2016; Dordevic et al., 2020). Against this background, it should become clear, that appropriate intervention strategies are needed to prevent direct disposal of expired but still edible food and to promote consumers’ willingness to consume such food. Furthermore, in order to develop a more comprehensive intervention approach aimed at reducing household food waste in industrialized countries such as Germany, it is essential that intervention strategies take into account the diverse behavioral contexts in which consumers engage with expired food in their everyday lives. Given that food consumption frequently occurs in social settings (e.g., family meals), an exclusive focus on fostering consumers’ willingness to personally consume expired but still edible food appears overly narrow (see Section 1.2 for further details). Accordingly, it is important to recognize the need for intervention strategies that also encourage consumers to offer expired but still edible food to others, alongside promoting their own consumption of such food. In pursuing both objectives, previous research on the psychological predictors of consumers’ willingness to consume expired but still edible food offers a valuable foundation for the development of effective interventions.

### 1.1 Psychological predictors of consumers’ willingness to consume expired but still edible food

**Figure 1:**
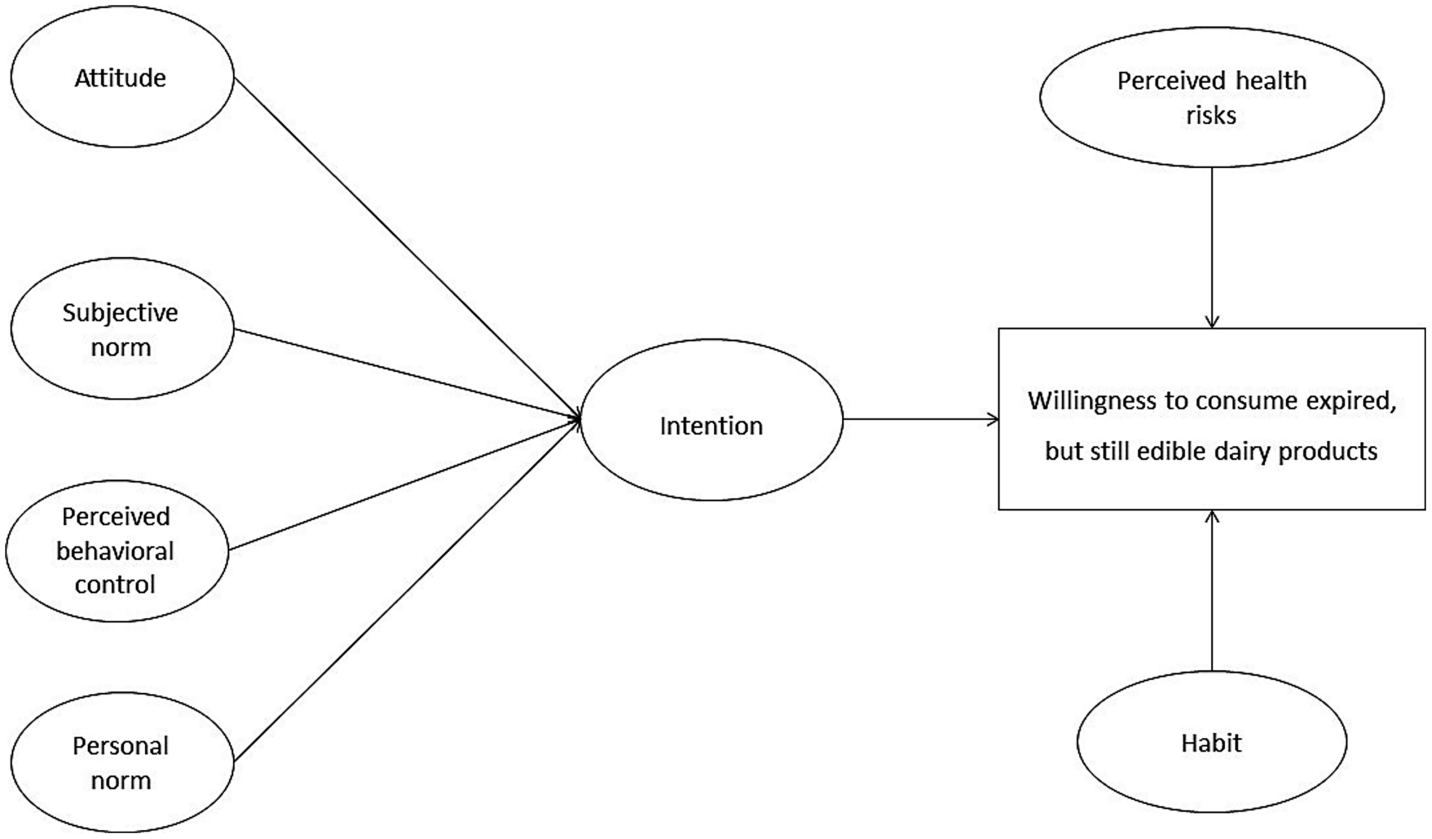
A comprehensive psychological model explaining consumers’ willingness to consume expired but still edible food proposed by Schmidt (2019).

In previous research on predictors of consumers’ willingness to consume expired but still edible food, two major categories of predictors exist: On the one hand, previous research has identified non-psychological predictors that affect consumers’ willingness to consume such food. For example, predictors that are related to consumers’ sociodemographic features, to the specific food category in question, or to package size and other product features belong to this category (see, e.g., Hooge et al., 2017; Thompson et al., 2018; Wilson et al., 2017). On the other hand, previous research has also identified a range of psychological predictors of consumers’ willingness to consume expired but still edible food (see e. g., Vittuari et al., 2023; Hebrok and Boks, 2017; Principato et al., 2021 for an overview). In this context, Schmidt (2019) provided empirical evidence of a comprehensive psychological model – as illustrated in Figure 1 – explaining consumers’ willingness to consume expired but still edible food based on an extended version of the *Theory of Planned Behavior* (TPB; see, e.g., Ajzen, 1991, 2011). The TPB is the theoretical model most often used in decision-making research in environmental psychology (see, e.g., Steg and Norlund, 2012 for an overview) as well as in food waste research (see, e.g., Stefan et al., 2013; Visschers et al., 2016; Graham-Rowe et al., 2015; Spence and Pidgeon, 2010; Stancu et al., 2016). The TPB represents the reasoned action approach in explaining people’s behavior, i.e., the TPB best applies to behaviors that are deliberately performed (Klöckner, 2015). With regard to the TPB-based core of her comprehensive psychological model, following Schmidt (2019), consumers’ willingness to consume expired but still edible food is directly affected by their intention to consume expired but still edible food. This intention, in turn, represent a reasoned choice that people make by weighing up various upstream predictors: This includes consumers’ attitude toward the consumptions of expired but still edible food (i.e., the extent to which engaging in the consumption of expired but still edible food is positively or negatively evaluated). Furthermore, consumers’ subjective norms (i.e., the extent to which the person believes that important others would approve or disapprove of the consumption of expired but still edible food) are considered. Finally, consumers’ intention is affected by their perceived behavioral control (PBC; i.e., perceived limits of resources, abilities, or opportunities to consume expired but still edible food; see, e.g., Klöckner, 2015 for an overview).

As mentioned above, the TPB is best suited to explain individuals’ deliberate behavioral decisions, which are based on a reasoned evaluation of the advantages and disadvantages of performing a specific behavior. While consumers’ willingness – or their decision – to consume expired but still edible food is certainly rooted in such a rational decision-making process, the nature of this behavior suggests that additional influencing factors may also be at play. To account for this, Schmidt (2019) extended the TPB model by incorporating several additional constructs. Notably, she integrated the concept of consumers’ *personal norm –* specifically, the moral obligation to avoid the direct disposal of expired food. In environmental psychology, the *Norm-Activation Model* (NAM; Schwartz and Howard, 1981) offers another widely recognized explanatory framework, particularly suited to behaviors strongly influenced by moral considerations. For such behaviors, rational-choice models like the TPB may prove insufficient. Given that consuming expired but still edible food can significantly reduce household food waste (as described in Section 1), moral considerations should play an important role in shaping behavior – alongside rational cost–benefit assessments. Against this backdrop, incorporating the NAM into models explaining consumers’ willingness to consume expired but still edible food appears highly appropriate. Within the NAM, personal norms are considered the ultimate determinants of an individual’s pro-environmental behavior. However, personal norms are assumed to influence behavior only when they are activated (Klöckner, 2015). This activation requires the individual to exhibit sufficient levels of the following predictors: (a) Awareness of need (e.g., recognizing that reducing household food waste helps mitigate global environmental problems such as climate change), (b) Awareness of consequences (e.g., understanding that reducing household food waste can effectively lower global food waste and related environmental impacts such as climate change), and (c) Ascription of responsibility (e.g., acknowledging personal responsibility for contributing to or preventing negative environmental consequences). Although Schmidt (2019) did not explicitly include these three variables as separate constructs in her model, they are indirectly represented through the inclusion of personal norms.^1^

In addition to further addressing moral considerations, Schmidt (2019) also acknowledged that consumers frequently face decisions about expired food in their daily lives. Thereby, these decisions should be often made under similar situational conditions and, thus, may become routinized. To capture this, she included the construct of *habit* – specifically, the habit of directly discarding expired food – in her model. According to Klöckner and Verplanken (2012, p. 198), “habits are defined as cognitive structures automatically determine future behavior by linking specific situational cues to (chains of) behavioral patterns.” Prior research in environmental psychology provides strong empirical support for the idea that habits are significant (often inhibitory) predictors of everyday pro-environmental behaviors. Therefore, to more accurately explain such behaviors, constructs like habits are often added to models such as the TPB or NAM (see Klöckner, 2015, for a comprehensive overview). Schmidt (2019) followed this approach in her own model explaining consumers’ willingness to consume expired but still edible food.

As already mentioned in Section 1, consumers frequently report significant confusion regarding the meanings and implications of different date labels when deciding whether to consume or dispose of expired but possibly still edible food. Since this confusion primarily concerns the perceived edibility of the food, it consequently has a strong influence on consumers’ perceived health risks associated with consuming such products (see, e.g., Visschers et al., 2016). In light of this, Schmidt (2019) ultimately integrated perceived health risks into her model as a barrier directly linked to the behavior in question. Specifically, she implied perceived health risks when consuming expired but still edible food as a relevant factor that can hinder consumers’ willingness to consume such food.

### 1.2 Extending previous research perspectives: consumers’ willingness to consume expired but still edible food versus their willingness to offer such food to others

Schmidt’s (2019) psychological model offers a solid theoretical foundation for understanding consumers’ willingness to consume expired but still edible food. It also serves as a useful basis for designing effective intervention strategies to encourage this behavior. However, despite its comprehensive approach, the model highlights the need for further expansion of research perspectives. Such expanded research perspectives can, for example, refer to different types of *behavioral contexts* in which consumers’ consumption practices with respect to expired food can take place in their daily lives. Since food consumption often takes place in social contexts (e.g., family meals, bringing food to a party or picnic with friends, offering snacks to guests), focusing only on consumers’ willingness to consume expired but still edible food themselves seems too short-sighted. Thus, it seems necessary to extend previous research perspectives by also considering consumers’ willingness to offer expired but still edible food to others.

Although it seems likely that consumers’ willingness to offer expired but still edible food to others is influenced by the same factors that predict their own consumption, socially determined contexts introduce additional complexity. Explaining people’s consumption practices in such contexts requires consideration of further relevant predictors. At the very least, it may involve different levels of influence for some of the already identified predictors. In this context, consumers’ *good provider identity* (i.e., consumers’ desire to “purchase and prepare sufficient amounts of food so that family members and guests are well catered for,” Visschers et al., 2016, p. 68; see also, e.g., Graham-Rowe et al., 2015; Porpino et al., 2016 for further information) should strongly affect their willingness to offer expired but still edible food to others in addition to the other predictors already considered for their own willingness to consume such food. Thereby, consumers’ desire to be a good provider refers not only to the amount of food but also to the quality of food, thus, to provide healthy and tasty foods to family members and guests (Evans, 2011; Graham-Rowe et al., 2014; Hebrok and Boks, 2017). As mentioned by Aschemann-Witzel et al. (2020, p. 586) “it represents an identity because the resulting behavior is driven by an ideal role that many consumers aim to fulfill, and this identity motivates certain food choices and handling practices, including decisions to dispose of food.”

In addition to consumers’ good provider identity, which likely plays a particularly important role in their willingness to offer expired but still edible food, subjective norms may also represent an even stronger predictor in this context. Compared to their own consumption, consumers’ willingness to offer such food to others may be more heavily influenced by perceived social expectations and the approval of others. Following the results presented by Schmidt (2019), consumers’ subjective norms were shown to be significant, but not the strongest predictors for their intention to consume expired food: The data analysis revealed stronger effects for consumers’ personal norm as well as for their perceived behavioral control to prevent direct disposal of expired food for their intention. But as mentioned above, offering (expired) food to others represents an action which is performed in a clearly more social context as when consumers have to decide about the own consumption of expired food. Thus, considerations about the expectations as well as about the own consumption-choices of (important) others, should be of higher importance for consumers’ intentions to offer expired but still food as it was shown for their own consumption.

Taken together, considering additional predictors such as consumers’ good provider identity and the likely greater importance of subjective norms in social contexts, it seems reasonable to assume that offering expired but still edible food to others is a more challenging behavior. In other words, it is likely influenced by more or stronger behavioral barriers compared to consuming expired but still edible food oneself. Therefore, the following research hypothesis was formulated for the present study:

*H0*: Participants report a significantly higher willingness to consume expired but still edible food than they report for offering expired but still edible food to others.

### 1.3 Theory-based selection of appropriate intervention techniques for the promotion of consumers’ willingness to consume and to offer expired but still edible food

Environmental psychological research and intervention practices provide a comprehensive pool of possible intervention techniques that can be used to promote people’s engagement in a range of pro-environmental behaviors (see, e.g., Steg et al., 2012a,b; Abrahamse, 2019; Abrahamse et al., 2005 for an overview). Thus, there are numerous opportunities to promote consumers’ willingness to both consume and offer expired but still edible food by applying environmental psychological intervention techniques. To maximize the effectiveness of these interventions, the focus should be on techniques that directly influence the key psychological predictors of consumers’ willingness to engage in these behaviors. With regard to the comprehensive explanation model described above, diverse intervention techniques focusing on the model’s specific predictors can be inferred. In this context, considering the provision of information – representing the most often used intervention technique in environmental psychological research (see e.g., Abrahamse and Matthies, 2012) – can be an effective intervention technique targeting several of the model’s predictors.

In environmental psychological research, intervention studies providing information to their target audience are generally aimed at changing psychological predictors for peoples’ engaging in pro-environmental behaviors, such as their knowledge, awareness or perceived/personal norms. In this context, two types of provided information can be differentiated: On the one hand, information about environmental problems – i.e., *problem knowledge* referring to the existence and extent of an environmental problem (like the climate crisis and the emissions resulting from global food production and consumption). Additionally, problem knowledge interventions can include information about the individual’s own contribution to these problems (for example, the CO₂ emissions generated by household food waste). On the other hand, information-provision interventions can (also) provide information about effective actions that can be taken by consumers to alleviate these problems (i.e., *action knowledge* on food waste-preventing behaviors referring to expired food; Abrahamse and Matthies, 2012). Against this background and specifically referring to the aim of promoting consumers’ willingness to consume and to offer expired but still edible food, both types of information-provision interventions seem to be appropriate. In the following, we use the term *informational intervention* to describe an approach that combines two key elements: First, it provides problem knowledge about the environmental issue of global climate change, with a focus on household food waste and consumers’ daily behaviors related to expired food. Second, it offers action knowledge about food waste–preventing behaviors specifically concerning expired food, enabling consumers to effectively reduce household food waste.

With regard to the above-described psychological predictors of consumers’ willingness to consume and offer expired but still edible food, providing specific problem knowledge about global climate change (and specifically about household food waste and consumers’ daily behaviors affecting this issue) can be an effective intervention technique: This problem knowledge can activate and promote consumers’ feelings of moral obligation, strengthening their personal norms to prevent household food waste and encourage them to consume or offer expired but still edible food. With respect to the predictors relevant for activating personal norms as conceptualized in the NAM (see Section 1.2 for details), this intervention effect is expected to occur through its direct impact on consumers’ awareness of need, awareness of consequences, and ascription of responsibility, all grounded in the problem-related knowledge provided. Against this background, the following research hypothesis was formulated for the present study:

*H1a*: Participants receiving an informational intervention (i.e., EG participants) report a significantly higher personal norm for the consumption of expired but still edible food than participants who receive a placebo intervention do (i.e., CG participants).

Additionally, the provided action knowledge – which highlights that expired food does not need to be discarded immediately and offers information about the typical edibility periods of various expired foods – should help reduce consumers’ perceived health risks associated with consuming expired but still edible food. That is why the following research hypothesis was formulated for the present study:

*H1b*: EG participants report significantly lower perceived health risks when consuming expired but still edible food than CG participants do.

Thinking ahead and taken together these assumptions of the direct effects of such an informational intervention on relevant predictors for consumers’ willingness to consume and to offer expired but still edible food, we further expect this willingness to be affected by the informational intervention at all. Therefore, these final research hypotheses were formulated:

*H2a*: EG participants report a significantly greater willingness to consume expired but still edible food than CG participants do.

*H2b*: EG participants report significantly greater willingness to offer expired but still edible food to others than CG participants do.

In order to provide a more comprehensive intervention evaluation procedure in the present study, we finally explored the assumed mediation processes: These processes refer to the intervention’s effects on consumers’ willingness to consume or offer expired but still edible food. We proposed that this effect is mediated by the intervention’s impact on consumers’ personal norm and their perceived health risks when consuming expired but still edible food.

Taken together, the main objective of the present study was to evaluate the effects of an informational intervention on consumers’ willingness to consume and to offer expired but still edible food as well as the psychological mechanisms underlying these effects. By doing this, the present study aims to provide initial insights into possible effective intervention approaches. These approaches could encourage consumers to consider consuming expired food in the first place, rather than disposing of it immediately after the best-before date has passed.

## 2 Materials and methods

### 2.1 Data collection and participants

We administered an online survey developed with the SoSci survey software in September 2019. Participants were recruited from the SoSci Panel. This panel represents a large pool of mostly highly educated volunteer respondents from German-speaking countries (mostly from Germany; for additional information, see Leiner, 2016). Altogether, 819 people took part in the online survey, whereas 660 people completed the entire survey. With regard to data quality, we excluded unreliable cases by excluding participants, who (a) reported to (nearly) never be responsible for handling different types of food in their household (*N* = 645; see Section 2.2 and Appendix Table A1 for details on these control variables); (b) were not at least 18 years old (*N* = 643); (c) report to (nearly) never buy dairy products for their household (*N* = 617; see again Section 2.2 and Appendix Table A1 for details on these control variables). We further excluded all participants, who did not provide an appropriate answer to the attention check-question, which was asked following the informational intervention in the experimental group as well as the placebo informational intervention in the control group (*N* = 584; see Section 2.2 for details)^2^. Finally, we decided to exclude all participants, who completed the entire online-survey in less than 10 min, as this comparatively short duration of answering the entire survey made it unlikely that the content of the survey would be adequately addressed by these participants (*N* = 558).^3^ After this whole procedure of excluding unreliable cases from the data, 558 people formed the final sample (281 EG participants and 277 CG participants).

This final sample contained more women (62.7%) than men (36.1%), whereas 1.3% of the participants defined themselves otherwise. Participants’ ages ranged from 18 to 82 years (*M* = 41.04, *SD* = 15.25). As expected, the final sample was highly educated (with 64.7% of the participants reporting a university degree and 16.4% reporting a general higher education entrance qualification). Most of the participants reported that they were employed (48.9%), studying (16.5%), or retired (12.2%). Household income ranged from less than 800€ (11.2%) to more than 5,000€ (8.9%) per month. Table 1 provides an overview of participants’ sociodemographic features.

**Table 1:** Sociodemographic features of the sample (*N* = 558).

Sociodemographic feature	Sample (%)
Gender	
Male	36.1
Female	62.7
Otherwise	1.3
Age	
18–25	17.6
26–40	37.7
41–60	32.7
61–65	5.5
>66	6.5
Education	
Completed primary school	0.5
Secondary education	17.0
Higher education entrance qualification	16.4
University degree	64.7
Household income	
Less than € 800	11.2
€ 801 – € 1,500	14.9
€ 1,501 – € 2000	10.6
€ 2001 – € 2,500	12.7
€ 2,501 – € 3,000	10.2
€ 3,001 – € 3,500	11.0
€ 3,501 – € 4,000	8.9
€ 4,001 – € 5,000	11.5
More than € 5,000	8.9
Average household size (SD)	2.32 (1.20)

### 2.2 Study procedure

At the beginning of the online survey, participants were asked for some control variables (e.g., participants’ responsibility for handling different types of food in their household) in order to exclude inappropriate participants/unreliable cases from the analyses. Furthermore, participants were asked a large variety of questions, which were later used for the randomization check procedure between EG and CG participants (i.e., questions about the importance of sustainability-, health- and economy-related aspects of participants’ food consumption practices in general, about how frequently dairy foods are consumed in their household in a typical month, and how often they make decisions about the edibility of dairy foods on the basis of the best-before date labels; see Appendix Table A1 for a full overview of all questions/items used in the present study).

After these initial questions, participants were randomly assigned to the informational intervention (EG participants) or the placebo intervention (CG participants; see Section 3.3 for details). Following the informational/placebo intervention, EG and CG participants were asked an attention-check question referring to the presented information per group: “*Please briefly name two tips on climate-friendly food consumption [on wholesome food consumption and drinking] that you found particularly interesting or particularly suitable for you/your household*.” The two selected tips were to be entered by the participants in two open answer fields (see Appendix Table A2 for details), whereby keywords were also sufficient. The EG and CG participants’ entries were checked after data collection to ensure that they matched the information presented for each group. All participants whose entries did not relate to the information presented in their group were excluded from the data analysis. This includes cases where tips were given that were not included in the intervention (e.g., plastic avoidance as a relevant tip in the EG), no tips were named, or statements such as “all tips” were made.

Following the attention-check question per group, the survey continued for both groups with the measurement of participants’ willingness to consume expired but still edible food as well as their willingness to offer such food to others in a hypothetical food-choice experiment. Afterwards, a diverse array of psychological predictors determining participants’ willingness to consume or offer expired but still edible food was measured. This was done to capture more relevant variables for the randomization check and to examine research hypotheses H1a and H1b. At the end of the survey, participants were asked for relevant sociodemographic features.

#### 2.2.1 Measuring participants’ willingness to consume and to offer expired but still edible food

As mentioned above, participants’ willingness to consume expired but still edible food as well as their willingness to offer such food to others was measured by using a hypothetical food-choice experiment. By doing so, we used nearly the same experimental procedure as the one used by Schmidt (2019).

Thus, the hypothetical food-choice experiment in the present study was designed for dairy products (i.e., for yogurt and—to extend Schmidt’s (2019) experimental procedure—also for cheese). We decided to focus on dairy foods for the same reasons described by Schmidt (2019): (1) Conventionally produced dairy foods are causing high climate emissions (e.g., with conventionally produced yogurt causing 1.7 kg CO_2_ equivalents per kg and conventionally produced cheese causing 5.7 kg CO_2_ equivalents per kg; Reinhardt et al., 2020), (2) Dairy products are still characterized by high consumption levels in Germany (122.02 kg per capita in 2022; Federal Agency for Agriculture and Food, 2023). (3) Previous research has implied that date labels (especially the best-before label) are highly relevant for consumers’ edibility decisions, especially with respect to dairy products (WRAP, 2015; Thompson et al., 2018; WRAP, 2013).

**Figure 2:**
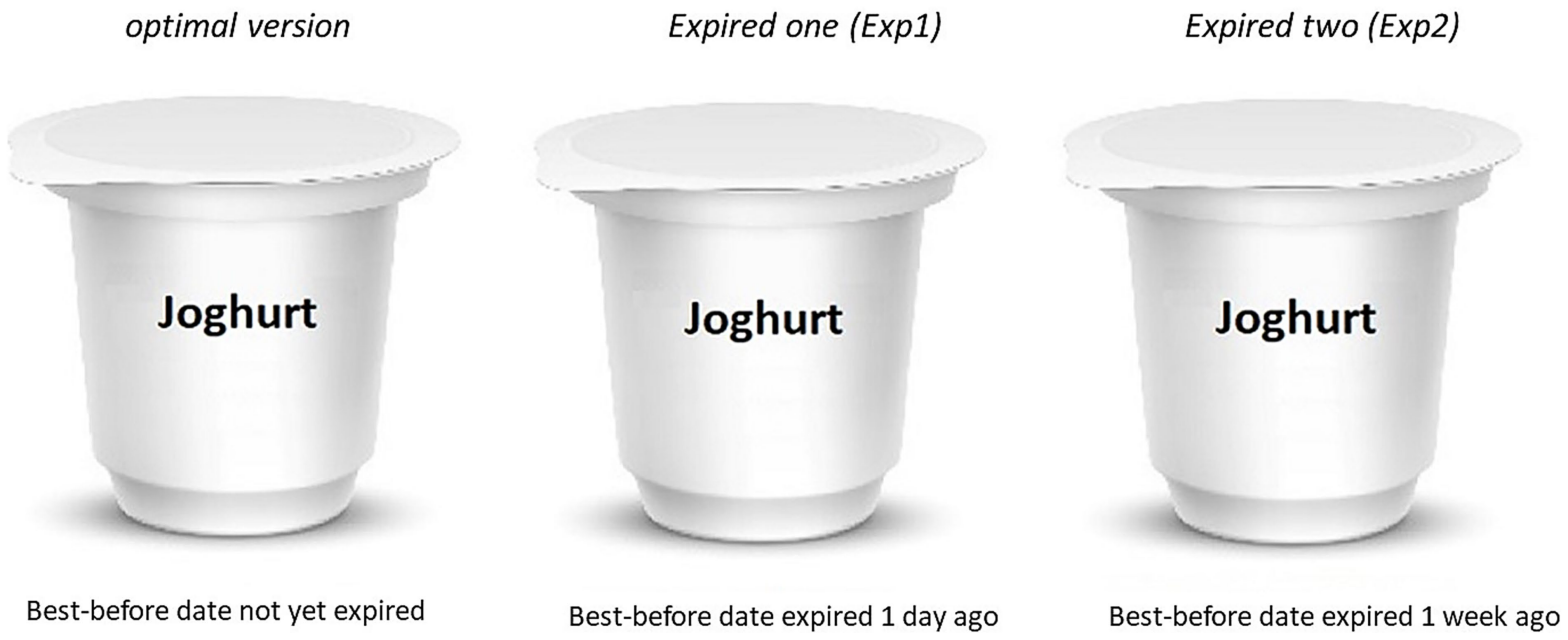
The neutrally designed yogurt with varying expiration dates used in the hypothetical food-choice experiment.

**Figure 3:**
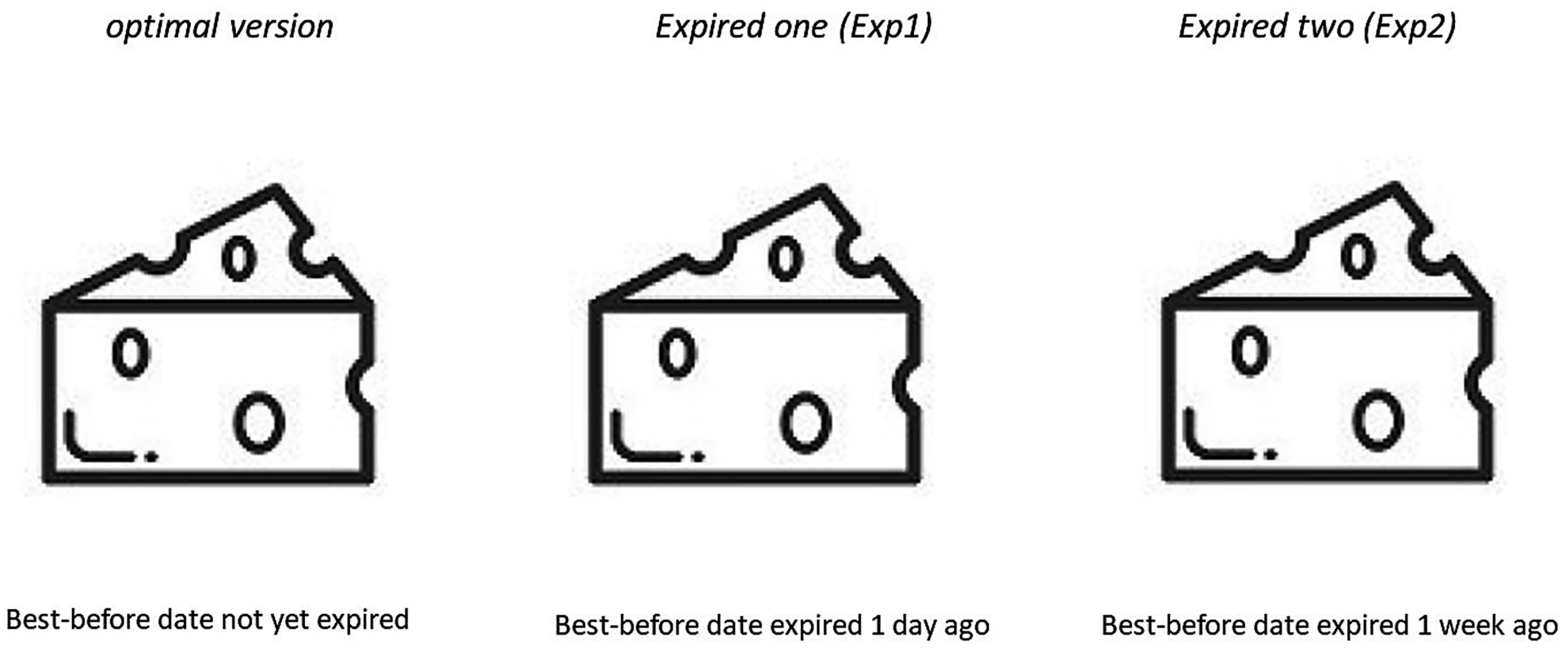
The neutrally designed cheese with varying expiration dates used in the hypothetical food-choice experiment.

In the food-choice experiment, based on the materials used by Schmidt (2019), a neutrally designed yogurt (see Figure 2) as well as a neutrally designed cheese (see Figure 3) with varying expiration (best-before) dates was shown. Thus, we used three versions of the same yogurt/cheese across the entire experimental procedure: (1) one version showing an unexpired yogurt/cheese (*optimal version*), (2) another version showing yogurt/cheese 1 day beyond the best-before date [*expired one* (*Exp1*)], and (3) another version showing yogurt/cheese 1 week beyond the best-before date [*expired two* (*Exp2*)]. We chose these three variations of expiration dates in order to vary the perceived difficulty of the choice-options (see Appendix Table A4 for an overview on the distribution of frequency of each choice per choice-set in the whole experiment): Thereby, we expected the choice of the optimal version to be characterized by the lowest difficulty-level, the choice of the Exp1-option to be characterized by a middle difficulty-level (due to the short period of time since the best-before date has passed) and the Exp2-option to be characterized by the highest difficulty-level (due to the comparatively long period of time since the best-before date has passed). As already mentioned in Section 1, for both time periods since the best-before date has passed, the edibility (and thus no justified health risks) of dairy products like yogurt and cheese can generally be assumed (see, e.g., Verbraucherzentrale Hamburg, 2019 for examples). This is especially the case since appropriate storage of all food options in the fridge was mentioned in the instructions of each choice set (see below for details).

**Figure 4:**
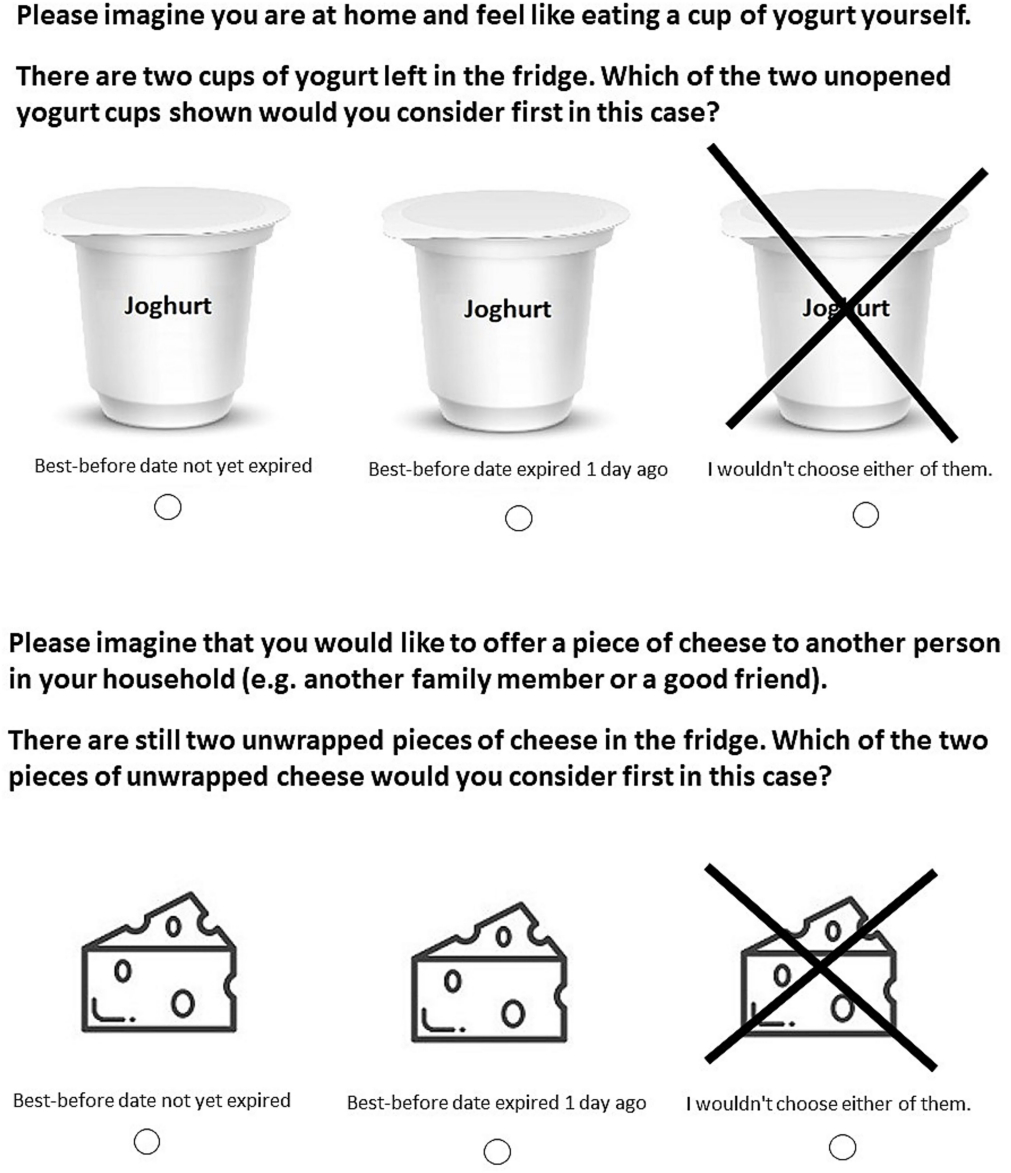
Examples of the choice sets presented in the hypothetical food-choice experiment.

In the experiment, there were six choice sets (three per yogurt and three per cheese) in which participants were asked to “*Imagine that you are at home, ready to select a yogurt. There are still two unopened yogurts [unwrapped pieces of cheese] in the fridge. Which one of the presented, unopened yogurts [which of the two pieces of unwrapped cheese] would you choose to consume?*.” Additionally, we integrated six additional choice sets (again, three per yogurt/cheese) in which participants were asked to “*Imagine that you are at home with friends and you want to offer them a yogurt. Which one of the presented, unopened yogurts [which of the two pieces of unwrapped cheese] would you choose to offer?*.” In each choice set, participants saw two out of the three versions per product (yogurt/cheese) in randomized positions. Across the entire experimental procedure for the yogurt/cheese, participants had to choose between the optimal version and Exp1, between the optimal version and Exp2, and between Exp1 and Exp2. In every choice set, participants also had the option “*I would not choose either of them.*” (see Figure 4 for examples of the choice sets that were used).

In the analysis, an overall score representing participants’ willingness to consume expired but still edible food was calculated. Another overall score representing their willingness to offer expired but still edible food to others was also calculated. Both scores were computed as the mean values of the six choice sets referring to participants’ own consumption or willingness to offer. Higher values for each score represent a higher willingness to consume or offer expired but still edible food.^4^

Although Schmidt (2019) used the calculated overall choice score per participant as a continuous variable, we decided to treat both calculated choice scores in the present study as categorical variables. For this reason, we chose to use appropriate non-parametric test procedures in the data analyses when examining these dependent variables (see Section 3.2).

#### 2.2.2 Measuring psychological predictors of consumers’ willingness to consume expired but still edible food

We measured the psychological predictors of participants’ willingness to consume expired but still edible food with items/scales taken/adapted from previous studies in the same or in a comparable research field. Except for the items used to capture participants’ attitudes toward the consumption of expired but still edible food, all items were measured on a six-point Likert scale ranging from 1 (*do not agree at all*) to 6 (*completely agree*), introduced by the following question: “*To what extent do you agree with the following statements?*” (see Appendix Table A1 for a complete overview of all items/scales used in the present study).

A middle category was deliberately omitted for these items/scales in order to prevent response bias in the sense of a *tendency toward the middle* among the participants (see, e.g., Rost et al., 1999; Bortz and Döring, 2006 for an overview). In order to avoid forcing the participants to express an opinion by not using the middle category if there was really no clear response tendency, an alternative response-option (“*no answer*”) could be selected for all items used in the present study.

##### 2.2.2.1 Attitudes toward the consumption of expired but still edible food

Based on the items used by Schmidt (2019) and Stancu et al. (2016), participants’ attitudes toward the consumption of expired but still edible food were measured with three items, which were answered on a 6-point Likert scale (e.g., ranging from 1 = *not completely negative* to 6 = *very negative*). All items were introduced by the following sentence: (“*I find that using up expired but still edible food in my household is …”*). With *α* = 0.89, this scale demonstrated very good reliability (Field, 2013; Gliem and Gliem, 2003).

##### 2.2.2.2 Subjective norms for the consumption of expired but still edible food

We adapted three items from Klöckner and Blöbaum (2010) and Schmidt (2019) to measure participants’ subjective norms [e.g., “*People who are important to me (*e.g.*, family and friends) expect me to consume expired but still edible food in my household”*], resulting in a scale with an acceptable level of reliability (*α* = 0.69).

##### 2.2.2.3 Perceived behavioral control (PBC)

Participants’ PBC was measured with three items (e.g., “*I can think of* var*ious ways that I can consume expired but still edible food in my household”*). With *α* = 0.68, this scale also demonstrated acceptable reliability. These items were taken from Schmidt (2016, 2019) and from Visschers et al. (2016).

##### 2.2.2.4 Personal norm for the consumption of expired but still edible food

Participants’ personal norms for the consumption of expired but still edible food were measured with two items adapted from Schmidt (2016, 2019), e.g., “*No matter what other people think or do, due to my values/principles, I feel obliged to consume expired but still edible food in my household*.” This scale demonstrated very good reliability (*r_Spearman_* = 0.71, *p* < 0.001).

##### 2.2.2.5 Perceived health risks when consuming expired dairy food

Perceived health risks when consuming expired dairy food were measured with three items taken from Schmidt (2019) and from Visschers et al. (2016), e.g., “*I think eating dairy products that expired some days ago is completely harmless*.” The resulting scale demonstrated good reliability (*α* = 0.74).

#### 2.2.3 Additional variables captured for the randomization check

In order to provide comprehensive data for the randomization check between EG and CG participants, we measured some additional variables. We chose variables that were likely to determine whether participants would be willing to offer expired but still edible food to others (i.e., participants’ good provider identity; see Section 1.2 for details) or that were relevant for consumers’ pro-environmental behaviors (for which their consumption practices with respect to expired food represent concrete examples; i.e., participants’ environmental attitude and their biospheric value orientations).

##### 2.2.3.1 Good provider identity with respect to expired food

Participants’ good provider identify with respect to expired food was measured with two items (e.g., “*I do not want other people in my household [family members, friends, guests, etc.] to eat expired food, even if it is still edible*”), which were newly created. The resulting scale demonstrated good reliability (*r_Spearman_* = 0.60, *p* < 0.001).

##### 2.2.3.2 Environmental attitude

Participants’ environmental attitude was measured with a short version of the New Environmental Paradigm (NEP; Dunlap, 2008; Dunlap et al., 2000). Our scale consisted of six items (e.g., “*We are approaching the limit of the number of people the earth can support*”) and showed good reliability (*α* = 0.73).

##### 2.2.3.3 Biospheric value orientation

We measured participants’ biospheric value orientation with four items adapted from Groot and de Groot and Steg (2008). The items were introduced by “*How much do you consider the following aspects to be guiding principles in your life? Preventing environmental pollution: protecting natural resources*” and answered on a 7-point Likert scale (ranging from 1 = *not important* to 7 = *very important*). With α = 0.86, the scale demonstrated very good reliability.

### 2.3 Implementation of the informational intervention

Following the randomized group-assignment procedure in the online survey, EG participants received the informational intervention. The intervention was introduced by a brief introductory text: “*In the following section of our survey, we would now like to present some selected recommendations for climate-friendly food consumption. Please read all the recommendations carefully and at your leisure*.”

On the subsequent survey page, participants in the experimental group (EG) were presented with a list of four recommendations for climate-friendly food consumption (see Appendix Table A2 for the full text). This list was introduced by a brief text providing problem knowledge about the environmental issue of global climate change and the significant role that food production and consumption patterns play in contributing to this problem: “*Climate protection tastes good! Tips for climate-friendly food consumption: The consequences of climate change are becoming more and more noticeable for all of us—an increase in storms and floods, droughts, and crop failures. In this context, our diet also contributes significantly to the greenhouse effect, especially through the production and processing of food—from cultivation to the kitchen. In Germany, food consumption accounts for around one fifth of the emissions of climate-impacting gasses. Thus, there are also many ways for private consumers to protect the climate when shopping and eating.”*

The list of recommendations concluded with a fourth recommendation that provided additional problem knowledge. This highlighted how household food waste contributes to climate change and how consumers’ daily consumption practices further influence household food waste. It also included action knowledge, emphasizing that expired food does not need to be discarded immediately and providing information about the typical edibility periods of various expired foods:

“*Avoid food waste! Every German throws away an average of 80 kg of food every year. Every discarded product is associated with the consumption of large amounts of energy, water, and other raw materials in the chain from cultivation to retail. Food waste also harms the climate: Avoidable food waste in the EU produces as much greenhouse gas per year as the Netherlands produce in total. Yet more than half of all household food waste could easily be avoided, for example, by planning meals and grocery shopping in advance or if food with expired best-before dates is not thrown away immediately or viewed as spoiled. For example, unopened yogurt stored in the fridge can still be used at least 1 week after the best-before date, a well-packaged piece of cheese can still be kept for up to 3 weeks, and unopened UHT milk even up to 8 weeks*.”

We deliberately provided EG participants with more information on climate-friendly food consumption than just on the issue of household food waste. This was done to avoid making the aim of the study—referring to the expected intervention effects – too obvious to the EG participants. By doing so, we aimed to prevent the disruptive effects of social desirability (see Section 5.3 for further considerations) on their food choices in the hypothetical food-choice experiment. In addition to addressing household food waste, we provided information on other types of climate-friendly food consumption that have received particular attention in previous research (i.e., reduction of meat and other animal-based food consumption, as well as the increased consumption of organic and seasonal, locally produced food; see, e.g., Abrahamse, 2019; Verain et al., 2015 for an overview).

Furthermore, a randomized order of the information and recommendations presented was deliberately avoided in the intervention. The aim was to make the recommendation regarding food waste particularly salient in participants’ perception and memory. Considering the well-established *recency effect* (i.e., a cognitive bias whereby individuals tend to better recall information presented most recently compared to earlier items in a list; see, e.g., Haugtvedt and Wegener, 1994), the food waste-related information was therefore intentionally placed at the end of the list of recommendations for each EG participant.

As mentioned above, CG participants received a placebo intervention in the online survey. They were presented with 10 recommendations on healthy nutrition (implemented with a comparable introduction and explanatory text providing several brief recommendations for a healthy diet; see again Appendix Table A2 for details). The recommendation on healthy nutrition were taken from the German Nutrition Society (Deutsche Gesellschaft für Ernährung e.V. (German Society for Nutrition), 2025).

## 3 Results

Data analyses was conducted by using SPSS (version 30) and the PROCESS-macro especially for the conducted mediation analysis. Within data analyses, levels of significance were interpreted as follows: *p* < 0.001 represented high significance, *p* ≤ 0.05 represented significance and *p* ≤ 0.08 represented marginally significance. All other *p*-values were interpreted as implying non-significant results. Unless otherwise reported, a confidence interval of 95.0% was defined in each analysis.

### 3.1 Preliminary analyses

#### 3.1.1 Willingness to consume versus to offer expired but still edible food

To examine the expected difference between participants’ willingness to consume expired but still edible food and their willingness to offer such food to others, we computed a Wilcoxon test with data from all participants (EG and CG participants).

In line with our expectations, the analysis revealed a significant and meaningful difference between the conditions (*z* = −16.268, *p* < 0.001, *d* = −1.90) with participants reporting significantly greater willingness to consume expired but still edible food themselves (*Mdn* = 2.00) than to offer such food to others (*Mdn* = 1.50). Thus, the data supported H0^5^.

#### 3.1.2 Randomization check

In order to ensure that significant differences in participants’ willingness to consume/offer expired but still edible food between EG and CG participants were not caused by relevant *a priori* group differences, we conducted an extensive randomization check. Thereby, we compared EG and CG participants on all the control variables, all the psychological predictors of their willingness to consume expired but still edible food (except for the variables for which an intervention effect was expected), all additional variables measured for the randomization check (see Section 2.2 for an overview), and participants’ sociodemographic features.

Out of these variables, we explored possible a priori group differences in metric variables by using a MANOVA, which showed no significant overall group-difference effect as well as no significant variable-specific group differences [*F*_(15)_ = 0.875, *p* = 0.59, *η_p_^2^* = 0.03; see Appendix Table A3 for an overview of all variable-specific comparisons]^6^. Furthermore, non-parametric tests for categorical and nominal variables also showed no significant group differences between EG and CG participants’ sociodemographic features (gender distribution: *χ*^2^ = 2.315, *p* = 0.31, *ω* = 0.065; education: *U* = 0.684; *p* = 0.49, *d* = 0.05). Taken together, this extensive randomization check procedure implied that there were no relevant a priori differences between EG and CG participants. Thus, any group differences identified in the dependent variables should be traced back to the informational intervention in the experimental group (in contrast to the placebo intervention in the control group).

### 3.2 Intervention evaluation procedure

In the first step of the intervention evaluation, we examined the effects of the informational intervention in the EG on the variables that were directly addressed by the intervention techniques we used (i.e., participants’ personal norm for the consumption of expired but still edible food and their perceived health risks when consuming expired food; see Section 1.3 for details).

In line with H1a, a *t*-*test* for independent samples showed that EG participants (*M_EG_* = 5.28, *SD_EG_* = 0.99) reported a significantly higher personal norm to consume expired but still edible food (*t_(552)_* = 1.805, *p* < 0.05, *d* = 0.15, *CI* [−0.015; 0.349]) than CG participants did (*M_CG_* = 5.11, *SD_CG_* = 1.18). By contrast and in line with H1b, another *t*-*test* for independent samples showed that EG participants (*M_EG_* = 2.04, *SD_EG_* = 1.08) reported significantly lower perceived health risks when consuming expired food (*t_(551)_* = −2.116, *p* < 0.05, *d* = −0.18, *CI* [−0.384; −0.014]) than CG participants did (*M_CG_* = 2.24, *SD_CG_* = 1.14). Taken together, these results clearly suggest that the informational intervention had the intended effect on both examined variables in the experimental group^7^.

To examine the intervention’s effects on participants’ willingness to consume and to offer expired but still edible food, we computed two *U-tests*. With regard to participants’ willingness to consume expired food, we found no significant group-difference (*z* = −1.647, *p* = 0.10, *d* = 0.14, *Mdn_EG_* = 2.00; *Mdn_CG_* = 2.00), while referring to participants’ willingness to offer expired food to others, we found a significant group-difference (*z* = −2.584, *p* < 0.05, *d* = 0.22) with EG participants reporting higher willingness to offer expired but still edible food (*Mdn_EG_* = 1.50) than CG participants did (*Mdn_CG_* = 1.33). Against this background, H2a was not supported, while H2b was supported by our data^8^.

**Figure 5:**
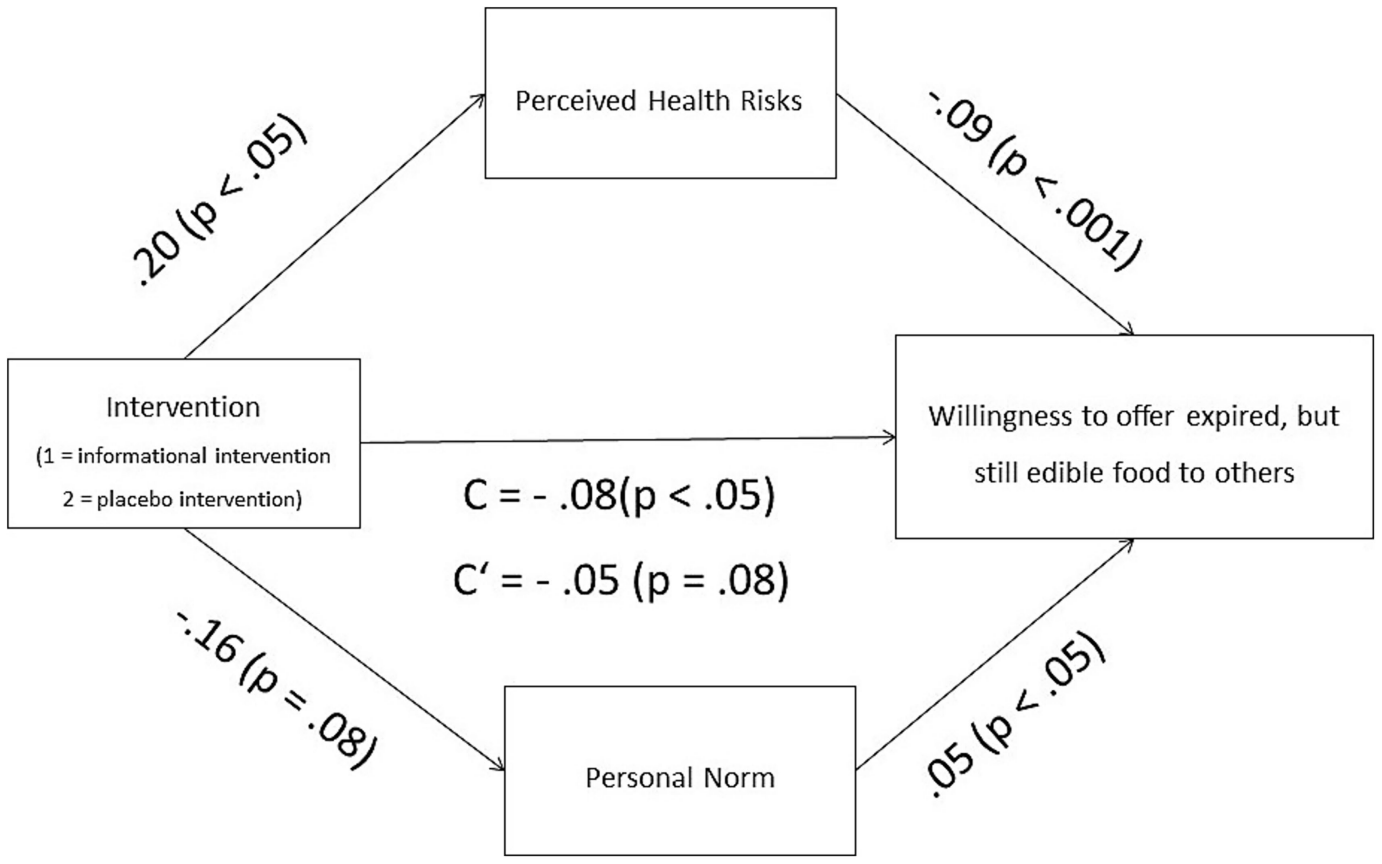
Results of the mediation analysis (conducted for participants’ willingness to offer expired but still edible food).

In order to provide a more comprehensive intervention evaluation procedure in the present study, we finally explored the assumed mediation processes. Since the data analysis identified a significant group-difference only referring to participants’ willingness to offer expired but still edible food to others, the mediation analysis was conducted for this dependent variable only: As summarized in Figure 5, within the analysis, an effect of the intervention (with lower values representing the informational intervention in the EG) on participants’ willingness to offer expired but still edible food was observed, *B* = −0.08, *p* < 0.05, *CI* [−0.017; −0.212]. After entering both mediators (personal norm and perceived health risks) into the model, the intervention predicted perceived health risks significantly (*B* = 0.20, *p* = 0.04, *CI* [0.014; 0.384]), which in turn predicted participants’ willingness to offer expired but still edible food highly significant (*B* = −0.09, *p* < 0.001, *CI* [−0.119; −0.067]). Furthermore, the intervention predicted participants’ personal norm marginally significant (*B* = −0.17; *p* = 0.07, *CI* [−0.349; 0.016]), which in turn predicted participants’ willingness to offer expired but still edible food highly significant (*B* = 0.05, *p* < 0.001, *CI* [0.019; 0.076]). Taken together, and in line with our theoretically inferred assumptions about the underlying mechanisms for the expected intervention effects on participants’ willingness to offer expired but still edible food to others, the mediation analysis provided important insights. The analysis implied that these intervention effects are mediated by participants’ personal norms and their perceived health risks (indirect effect personal norm = −0.008, *CI* [−0.018, −0.001]; indirect effect health risks = −0.018, *CI* [−0.0372, −0.001]).

## 4 Discussion

### 4.1 Summary and classification of the present findings in previous research

The main objective of the present study was to evaluate the effects of an informational intervention on consumers’ willingness to consume and to offer expired but still edible food. Additionally, the study aimed to examine the psychological mechanisms underlying these effects. By doing so, the present study intended to provide initial insights into possible effective intervention approaches. These approaches could encourage consumers to consider consuming expired food rather than disposing of it immediately after the best-before date has passed.

In order to provide a more comprehensive research perspective, the present study aimed to cover various behavioral contexts in which consumers engage in food waste-preventing consumption practices in their daily lives. Therefore, the study focused not only on consumers’ willingness to consume expired but still edible food themselves but also on their willingness to offer such food to others. In line with our expectations, the results showed that participants’ willingness to consume expired but still edible food themselves was significantly higher than their willingness to offer such food to others. To the best of our knowledge, no previous research has explicitly examined such differences in consumers’ food-waste-prevention behaviors involving expired food in different behavioral contexts as we did in the present study.

With regard to these initial findings, we evaluated our informational intervention by comprehensively considering participants’ willingness levels depending on their own consumption versus offering expired food to others. In the first step of the intervention evaluation, we examined direct intervention effects. Specifically, we focused on intervention-related changes in the psychological variables that our theoretical model suggested would be directly influenced by the intervention techniques. These variables included participants’ personal norm regarding the consumption of expired but still edible food, as well as their perceived health risks when consuming expired food. In line with our assumptions, our analyses identified a significantly higher personal norm and significantly lower perceived health risks for EG participants compared with CG participants who received a placebo intervention. Building on these findings, we went on to examine the hypothesized differences in participants’ willingness to consume and to offer expired but still edible food depending on the informational intervention in the EG. Our results showed that, compared with the CG participants, the EG participants were more willing to offer expired but still edible food to others, while there was no significant group-difference found for participants’ willingness to consume expired but still edible food themselves. In this context, it should be considered, that a *ceiling effect* (see, e.g., Ho and Yu, 2015; Wang et al., 2008) could be a reasonable explanation for the missing significant group-difference between EG and CG participants’ willingness to consume expired but still edible food: In contrast to participants’ willingness to offer expired but still edible food to others, our analysis showed a comparatively high willingness for the own consumption among all participants right from the start (see Section 3.1 for details). That is why the potential for a significant group-difference (i.e., a significant higher willingness for own consumption in the EG compared to CG) was much lower right from the start for this dependent variable than it could be assumed for participants’ willingness to offer expired but still edible food to others.

Irrespective of these considerations explaining the unexpected absence of an intervention effect on participants’ willingness to consume expired but still edible food themselves, the final mediation analysis was conducted to provide a more comprehensive intervention evaluation. This analysis suggested that the observed intervention effect on participants’ willingness to offer expired but still edible food was mediated by their personal norm and perceived health risks.

### 4.2 Implications for an intervention focusing on individuals’ daily consumption practices and on other relevant actors for (household) food waste-prevention

Considering all findings of the present study, it can be assumed that simple informational interventions, such as implemented in our study, have great potential for enacting relevant behavioral changes in the household-food-waste-prevention domain. In addition to the already existing intervention programs and campaigns that are designed to educate consumers about the concrete meanings of various date labels on food products (especially best-before vs. use-by dates, see, e.g., WRAP, 2023) in general, there is also a need for future interventions. There is an especially strong need for intervention techniques that can “act” more directly in the moment when consumers decide whether to consume or dispose of an expired but possibly still edible food product. This need aligns with the high relevance of habits in the direct disposal of expired food, as shown for example, by Schmidt (2019). Using appropriately designed prompt interventions could be a very promising approach for effective practical interventions. These prompts might include brief messages about typical edibility periods for a particular type of food or short instructions on how to assess a food product’s edibility through sensory testing. For an overview of prompt interventions (see, e.g., Steg and Norlund, 2012).

At the same time, however, this consideration also makes clear that the development and especially the implementation of most effective intervention techniques in this field can be realized only if efforts of diverse actors in the food consumption and production system are combined – thus, shifting the burden only to consumers should be considered as a quite inadequate perspective for intervention practice. This conclusion is easy to illustrate using the example of the above-proposed prompt intervention technique: Prompt interventions are typically most effective when the prompts are perceived as being as “close” as possible to the moment when consumers engage in the behavior in question. For example, deciding whether to consume or dispose of expired but still edible food. Therefore, food packaging seems to be the most appropriate location to place prompts aimed at preventing the direct disposal of expired but still edible food. Furthermore, it should be considered, that improving the accuracy of date labels (especially referring to best-before labels) could also represent an effective if not even more effective way for preventing household food waste. As, for example, implied by the WRAP online-guide (WRAP, 2019), intervention programs aiming to improve the accuracy of date labels and provide clearer communication to consumers generally require direct changes in conventional production processes. Therefore, although the household food waste domain – and especially consumers’ consumption practices involving expired food – should be viewed as private behavioral domains, change is needed not only in consumers’ daily consumption practices but also in the structural conditions and behavioral contexts in which these practices take place. Therefore, effectively reducing the amount of household food waste requires extensive changes in production and trade conditions. This also means changes in the relevant political measures that shape and promote current production and trade systems in the food sector. Only by combining the engagement of a wide range of actors in the food domain – such as consumers, traders and distributors, producers, and politicians – can these profound changes in our society be achieved. Only under these conditions can the United Nations’ target to halve per capita global food waste at the retail and consumer levels by 2030 be reached.

### 4.3 Limitations and implications for future research

Although the present study has important implications for intervention practices, there are nevertheless several limitations that should be considered to appropriately interpret the results.

As already mentioned in the Section 2.1, participants in the present study were recruited from the SoSci Panel, which represents a large pool of mostly highly educated volunteer respondents from German-speaking countries. Therefore, our findings cannot be generalized to the German population or to other populations without further research. The same limitation also applies to the generalization of our findings to the consumption of expired foods other than those examined here.

Additionally, there are some limitations involving the measures used in the present study: Since our results are based only on self-report measures, and thus, we cannot exclude the possibility that some inaccuracies and possible *response biases* could have negatively affected the data.

In addition to biases related to general changes in attention over the course of the survey –reflecting rather unconscious response biases, like *decision fatigue* (Baumeister et al., 2018) – biases that involve more consciously altered response behavior by participants should also be considered when interpreting the present results. Regarding the overall relevance of such biases in environmental psychological research (see Vesely and Klöckner, 2020 for an overview), *social desirability bias* in particular may have negatively influenced the data collected (see, e.g., Cerri et al., 2019 for details on this bias). Based solely on the data from the present study, we cannot say with absolute certainty that the identified intervention effects were not at least partly due to social desirability. Nevertheless, it should be noted that the present study did implement some measures aimed at fundamentally reducing or controlling for social desirability bias: Thereby, we gave explicit instructions in the survey to emphasize that no answers were considered right or wrong and that only participants’ personal opinions were relevant. Furthermore, we deliberately positioned the measurement of the central dependent variable – participants’ willingness to consume or offer expired but still edible food – before the measurement of the independent variables in the survey. This sequencing was chosen because the items measuring the independent variables specifically emphasized climate-friendly food consumption, food waste avoidance, and, naturally, willingness to consume expired food. Nevertheless, the exclusive use of self-report measures, along with the possibility that participants perceived subtle, differing cues about the researchers’ expectations during the interventions, remains a significant methodological limitation of the present study.

Some further limitations referring to the implementation of the informational vs. the placebo intervention in EG and CG should also be considered: Thereby, it should be mentioned, that the experimental manipulation differed in some aspects between the groups, what could have had affected *internal validity* of the present results in an unintended way. For example, although the lengths of the presented texts were tried to be comparable between EG and CG, it should be considered that the number of recommendations per intervention differed between both groups (with four recommendations presented in the EG and 10 recommendations presented in the CG). In order to exclude possible confounding effects of such methodological differences in intervention implementation, future studies should consider simplifying and equalizing the conditions.

Another methodological limitation of the present study refers to the hypothetical food-choice experiment used to capture participants’ willingness to consume and to offer expired but still edible food: Participants’ decisions in such hypothetical situations might not necessarily match the decisions they make in their real daily lives. Even if the question of comparability between participants’ choice/consumption behavior in the hypothetical food-choice experiment and in their real life cannot be definitively answered, the following aspects should be taken into account with regard to this limitation: On the one hand, it should be considered that we deliberately chose to measure the dependent variables in our study using a hypothetical choice experiment. This decision was made to increase the internal validity of our findings, due to the stronger control options for excluding confounding variables that such a measurement allows. On the other hand, a direct conclusion from participants’ choices in the hypothetical experiment to their actual choices in comparable situations in their everyday life was not so much the focus of the present study. In particular, as already mentioned before, our study was intended to provide initial insights into possible effective intervention approaches by which consumers can be encouraged to consider consuming/using expired food in the first place and not to dispose of it immediately after the best-before date has expired. That is, why in our food-choice experiment, participants should make their choice solely on the basis of a single product characteristic (i.e., based on the information about the expiration date per food-option). Referring to consumers’ actual consumption choices in real life, they should make these choices on the basis of a range of further criteria (especially referring to the foods’ sensory characteristics). But only if consumers are fundamentally motivated to take a closer look at the sensory characteristics of expired food, i.e., only if consumers are fundamentally willing to consume/use expired but still edible food at all, household food waste can finally be prevented. Nonetheless, focusing solely on internal validity and consumers’ fundamental willingness to consume or use expired but still edible food will not be sufficient. A truly comprehensive research perspective should go beyond this to identify effective ways to promote consumers’ food waste-preventing consumption practices referring to expired food. Therefore, future research should consider this limitation of the present study. For example, by using a comparable hypothetical food-choice experiment with improved *external validity* by integrating more information on the relevant characteristics of the presented food options. Or – ideally – future research should conduct appropriate real-life or even *field experiments* to examine consumers’ real-life consumption practices referring to expired food in addition to such hypothetical laboratory study designs: Regarding real-life experiments, studies capturing consumers’ actual willingness to consume expired but still edible food could be implemented. Furthermore, repeated measurements of participants’ willingness to consume expired but still edible food within a more longitudinal study design could be employed to specifically investigate possible long-term effects of interventions. Finally, implementing field experiments could further enhance the external validity of the findings, as field experiments capture not only real-life behaviors but also participants’ behavior in their natural, everyday contexts (see, e.g., Steg et al., 2012a,b for an overview).

Finally, a last limitation of the present study lies in the simplistic nature of the effect examined: Although, a very focused study design was deliberately chosen here in order to specifically analyze the effects of the implemented informational intervention on consumers’ willingness to consume and to offer expired but still edible food as well as the psychological mechanisms underlying these effects. However, such a reduced research design as a result also means that possible influences of other variables cannot be taken into account. That is why, the effects of other predictors for the examined dependent variables (as presented in Section 1.2) that could potentially act as moderators or further mediations for the examined intervention effects, could not be considered in the present study.

Taken together, it can be concluded that, in line with its overall research aim, the present study provides initial insights into possible effective intervention approaches. These approaches can encourage consumers to consider consuming or using expired food in the first place, rather than disposing of it immediately after the best-before date has expired. Therefore, the present study can make an important initial contribution toward more effective prevention of household food waste in Germany and beyond. Nonetheless, since there are several limitations that should be considered when interpreting the present findings, it should be clear that many research questions remain. These questions should be specifically addressed by future research in this field.

## Data Availability

The raw data supporting the conclusions of this article will be made available by the authors, without undue reservation.

## Footnotes

^1^In this context, it should be clarified, that a personal norm differs conceptually and also in its operationalization of an intention in the fact that a personal norm focuses on feelings of moral obligation to engage in a particular pro-environmental action (like the consumption of expired but still edible food), while an intention represent consumers’ specific purpose to engage in a particular pro-environmental action (Steg and Norlund, 2012). Although intentions and personal norms were highly correlated in the study by Schmidt (2019), data analysis showed that adequate discriminant validity was given for any of the examined constructs.

^2^The exclusion of participants based on this criterion was comparable between the experimental group and the control group (18 participants were excluded in the EG and 15 participants were excluded in the CG).

^3^Calculated across all participants, *M* = 1084.94 s (SD = 360.50 s).

^4^In each choice set, the higher score was assigned to the option with the longer expiration time. This resulted in the following numerical coding of participants’ choices: Optimal version (= 1) vs. Exp1 (= 2); optimal version (= 1) vs. Exp2 (= 2); Exp1 (= 1) vs. Exp2 (= 2). Consequently, the calculated overall scores representing participants’ willingness to consume and to offer expired but still edible food ranged between 1 and 2.

^5^A *post hoc* power analysis (G*Power, Faul et al., 2009) conducted for a Wilcoxon-Mann–Whitney test with two groups (*N*_EG_ = 281; *N*_CG_ = 277) implied the analysis would be sensitive to effects of *d* = 0.30 (implying small to medium effects; Cohen, 2013) with 86.2% power (*α* = 0.05, two-tailed).

^6^A *post hoc* power analysis (G*Power, Faul et al., 2009) implied, that the conducted MANOVA with two groups (*N*_EG_ = 207; *N*_CG_ = 197) would be sensitive to effects of *f* = 0.25 (implying medium effects; Cohen, 1988) with 91.3% power (*α* = 0.05).

^7^A *post hoc* power analysis (G*Power, Faul et al., 2009) conducted for a *t*-test for independent samples (*N*_EG_ = 279; *N*_CG_ = 274) implied the analysis would be sensitive to effects of *d* = 0.30 (implying small to medium effects; Cohen, 1988) with 94.1% power (*α* = 0.05, two-tailed).

^8^A *post hoc* power analysis (G*Power, Faul et al., 2009) conducted for a Wilcoxon-Mann–Whitney test with two groups (*N*_EG_ = 280; *N*_CG_ = 277) implied the analysis would be sensitive to effects of *d* = 0.30 (implying small to medium effects; Cohen, 1988) with 93.2% power (*α* = 0.05, two-tailed).
