# Supplementary material for: Simple and smart—promoting consumers’ willingness to consume and offer expired but still edible food through an informational intervention
Source: Front Psychol. 2025 Sep 12;16:1514312. doi: 10.3389/fpsyg.2025.1514312 (PMC12463826; doi:10.3389/fpsyg.2025.1514312)
Supplement: Supplementary file 1 [file Data_Sheet_1.PDF]

Appendix

Table A.1
Scales/ items used to measure psychological predictors of consumers' willingness to consume expired but still edible food as well as additional variables measured for randomization checks in the present study and descriptive statistics (calculated for the final sample *N* = 558).

**Scale/ items**		**Number of items**	**Formulation**	**Answer options**	***M* (*SD*)**	**Min**	**Max**
Control variables	Responsibility for handling different types of food in the household	5	How often are you responsible for the following actions in your household?<br>• Meal and grocery shopping planning<br>• Grocery shopping<br>• Food preparation<br>• Food storage<br>• Disposal of food waste	(almost) never; rarely; sometimes; often; (almost) always	4.26 (0.79)	1.00	5.00
	Importance of sustainability-, health-, and economy-related aspects of participants' food consumption practices in general	3	How much do you agree with the following statement?<br>• For me, it's important that the food I eat on a typical day have as little impact on the environment as possible.<br>• When I prepare/eat food, I think a lot about whether the food in question is completely harmless to my health or not.<br>• For me, it's important that the food I eat on a typical day is inexpensive.	do not agree at all - completely agree	3.98 (1.29)<br>4.45 (0.96)<br>3.26 (1.37)	1.00<br>1.00<br>1.00	6.00<br>6.00<br>6.00
	Dairy food consumption	1	How often are dairy products consumed in your household in a typical month? If you usually consume lactose-free dairy products or vegan/plant-based alternatives, please also include these in your answer.	(almost) never; 1-2 times per month; 2-3 times per month; 1-2	5.65 (0.77)	2.00	6.00

			times per week; 2-3 times per week; (almost) daily			
Use of expiration dates for consumption decisions referring to dairy foods	1	How do you usually decide whether dairy products in your household are still edible or should be disposed of?<br>• Product’s expiration date	(almost) never; rarely; sometimes; often; (almost) always	2.27 (1.18)	1.00	5.00
Attitudes toward the consumption of expired but still edible food	3	I find that using expired but still edible food in my household is...	not completely negative - very negative;<br>not completely unreasonable - very unreasonable;<br>not completely bad - very bad	5.68 (0.66)	1.00	6.00
Subjective norms for the consumption of expired but still edible food	3	People who are important to me (e.g., family and friends) expect that I consume expired but still edible food in my household.<br>People who are important to me (e.g., family and friends) try to consume expired but still edible food.<br>People who are important to me (e.g., family and friends) encourage me to consume expired but still edible food in my household.	do not agree at all - completely agree	4.05 (1.31)	1.00	6.00

Perceived behavioral control (PBC)	3	I can think of various ways that I can consume expired but still edible food in my household.<br>I don't find it difficult to use up expired but still edible food in my household myself.<br>I don't know how I can manage to use up food in my household that has expired but is still edible. (*)	do not agree at all - completely agree	5.29 (0.88)	1.00	6.00
Personal norms for the consumption of expired but still edible food	2	No matter what other people think or do, due to my values/principles, I feel obliged to consume expired but still edible food in my household.<br>I feel a strong personal obligation to consume expired but still edible food in my household.	do not agree at all - completely agree	5.19 (1.09)	1.00	6.00
Perceived health risks when consuming expired dairy food	3	I believe that the risk of becoming ill as a result of eating dairy products past its use-by date is high.<br>I think eating dairy products that expired some days ago is completely harmless. (*)<br>I am not concerned about the possible health risks of consuming expired dairy products. (*)	do not agree at all - completely agree	2.14 (1.11)	1.00	6.00
Good provider identity referring to expired food	2	I do not want other people in my household (family members, friends, guests, etc.) to eat expired food, even if it is still edible.<br>It would be unpleasant for me to offer other people in my household (other family members family members, friends, guests, etc.) expired food, even if it is still edible.	do not agree at all - completely agree	2.46 (1.39)	1.00	6.00

Environmental attitude/NEP	6	We are approaching the limit of the number of people the earth can support.<br>The natural balance is very sensitive and easy to disturb.<br>The earth is like a spaceship with very limited space and limited resources.<br>People are seriously abusing the environment.<br>If things continue as they are, we will soon experience a major ecological catastrophe.<br>The so-called “ecological crisis” humanity is facing is greatly exaggerated. (*)	do not agree at all - completely agree	4.93 (0.81)	1.00	6.00
Biospheric value orientation	4	How much do you consider the following aspects to be guiding principles in your life?<br>• Preventing environmental pollution: protecting natural resources<br>• Respect the earth: Harmony with other species<br>• Unity with nature: blending in with nature<br>• Protecting the environment: preserving nature	not important - very important	6.00 (0.97)	1.25	7.00

Note: Recoded items are marked with (*). *M* = mean value; *SD* = standard deviation; Min = Minimum; Max = Maximum.

Table A.2
Texts used for the informational intervention in the EG and for the placebo informational intervention in the CG and the corresponding attention-check questions

Text type	Text
Informational intervention in the EG	In the following section of our survey, we would now like to present some selected recommendations for climate-friendly food consumption. Please read all the recommendations carefully and at your leisure.<br>Climate protection tastes good! Tips for climate-friendly food consumption: The consequences of climate change are becoming more and more noticeable for all of us—an increase in storms and floods, droughts, and crop failures. In this context, our diet also contributes significantly to the greenhouse effect, especially through the production and processing of food—from cultivation to the kitchen. In Germany, food consumption accounts for around one fifth of the emissions from climate-impacting gases. Thus, there are also many ways for private consumers to protect the climate when shopping and eating.<br><br>Eat less meat and sausage!<br>The production of meat, sausage, and other animal-based foods, such as dairy products and eggs, is particularly energy-intensive and harmful to the climate. The production of 1 kg of beef releases around 28 times as many greenhouse gases as 1 kg of fruit or even 1,400 times as many as for 1 kg of vegetables. As is so often the case, less is more: So it's better to enjoy a Sunday roast with a clear conscience and instead draw on the diversity of the vegetable garden on other days—often without meat! Nutrition experts recommend eating eat small portions of meat two to three times a week.<br><br>Eat organic food—more and more often!<br>Compared with conventional agriculture, organic farming requires significantly less fossil energy, for example, from crude oil. Organic food not only protects the climate, it also has considerably fewer pesticide residues. In addition, organically farmed soils accumulate humus and can store greenhouse gases in it.

	Eat seasonal food from the region—instead of products that have traveled far!<br>Long-distance transportation by plane or truck requires a lot of energy and therefore contributes to climate change. They damage the environment and cause noise. Regional food generally has less of an impact on the climate if efficient means of transportation are used. Regional products can score particularly well in terms of climate protection and enjoyment when they are in season. Products from heated greenhouses produce up to 30 times more greenhouse gases than vegetables grown outdoors. The first choice is vegetables and fruit from the region and fresh from the field.<br><br>Avoid food waste!<br>Every German throws away an average of 80 kg of food every year. Every discarded product is associated with a high consumption of energy, water, and other raw materials in the chain from cultivation to retail. This waste also harms the climate: Avoidable food waste in the EU is responsible for as many greenhouse gases per year as the Netherlands as a whole. Yet more than half of all household food waste could easily be avoided, for example, by planning meals and grocery shopping in advance or if food with expired best-before dates is not thrown away immediately or viewed as spoiled. For example, unopened yogurt stored in the fridge can still be used at least 1 week after the best-before date has expired, a well-packaged piece of cheese can still be kept for up to 3 weeks, and unopened UHT milk even up to 8 weeks.
Attention-check in the EG	Please briefly name two tips for climate-friendly food consumption that you found particularly interesting or particularly suitable for you/your household (*answered in two open-answer fields*).
Placebo informational intervention in the CG	In the following section of our survey, we would now like to present some selected recommendations for climate-friendly food consumption. Please read all the recommendations carefully and at your leisure.<br>Ten tips for wholesome food and drink! Eating and drinking whole foods keeps you healthy and promotes performance and well-being. We have summarized how this can be achieved in the following 10 tips.<br>1. Enjoy a variety of foods<br>Take advantage of the variety of foods and eat a varied diet. Choose predominantly plant-based foods.<br>2. Eat vegetables and fruit—eat “5 a day”<br>Enjoy at least three portions of vegetables and two portions of fruit a day. The colorful selection also includes legumes such as lentils, chickpeas, and beans as well as (unsalted) nuts.

	3. Choose whole grains<br>When it comes to cereal products (e.g., bread, pasta, rice, and flour), whole grains are the best choice for your health.<br>4. Supplement your diet with animal foods<br>Eat milk and dairy products such as yogurt and cheese daily, fish once or twice a week. If you eat meat, then no more than 300 to 600 g per week.<br>5. Use healthy fats<br>Give preference to vegetable oils such as rapeseed oil and spreadable fats made from it. Avoid hidden fats. Fat is often “invisible” in processed foods such as sausage, baked goods, confectionery, fast food, and convenience products.<br>6. Cut down on sugar and salt<br>Foods and drinks sweetened with sugar are not recommended. Avoid them as much as possible and use sugar sparingly. Cut down on salt and reduce the proportion of salty foods. Season creatively with herbs and spices.<br>7. It is best to drink water<br>Drink around 1.5 l every day, preferably water or other calorie-free drinks such as unsweetened tea. Sugar-sweetened and alcoholic drinks are not recommended.<br>8. Prepare gently<br>Cook food for as long as necessary and as short a time as possible, using as little water and fat as possible. Avoid frying, grilling, baking, and deep-frying food.<br>9. Eat and enjoy mindfully<br>Give yourself a break for your meals and take your time when eating.<br>10. Watch your weight and keep moving<br>A balanced diet and physical activity go hand in hand. Not only is regular exercise helpful, but an active daily routine is helpful too, for example, walking or cycling more often.
Attention-check in the CG	Please briefly name two tips for wholesome food and drink that you found particularly interesting or particularly suitable for you/your household (*answered in two open-answer fields*).

Note: In both groups (EG and CG), the attention check-question was presented on a new page after the informational intervention/placebo intervention. Participants who did not name two of the presented tips (for climate-friendly food consumption/for wholesome food and drink)—at least by naming suitable keywords—were excluded from the final sample.

Table A.3
Overview of all variable-specific comparisons from the MANOVA with all metric variables used for the randomization check between the EG and CG participants.

**Variable**	**N_{EG}**	**M_{EG} (*SD*)**	**N_{CG}**	**M_{CG} (*SD*)**	***F***	***p***
Responsible for handling different types of food in their household	207	2.25 (1.15)	197	2.35 (1.16)	0.237	.63
Importance of health-related aspects referring to participants' food consumption practices in general		3.82 (1.48)		4.05 (1.41)	2.540	.11
Importance of sustainability-related aspects referring to participants' food consumption practices in general		4.25 (1.32)		4.10 (1.35)	1.188	.28
Importance of economy-related aspects referring to participants' food consumption practices in general		3.24 (1.38)		3.15 (1.40)	0.421	.52
Dairy food consumption		5.66 (0.79)		5.70 (0.63)	0.373	.54
Use of expiration dates for consumption decisions referring to dairy foods		4.26 (0.80)		4.23 (0.78)	0.669	.41
Attitudes toward the consumption of expired but still edible food		5.66 (0.63)		5.64 (0.67)	0.073	.79
Subjective norms for the consumption of expired but still edible food		3.92 (1.27)		4.05 (1.30)	1.026	.31
Perceived behavioral control (PBC)		5.34 (0.79)		5.19 (0.89)	2.963	.09
Good provider identity referring to expired food		2.39 (1.34)		2.50 (1.34)	0.728	.39
Environmental attitude/NEP		4.92 (0.78)		4.86 (0.83)	0.531	.47
Biospheric value orientation		5.95 (0.99)		5.92 (1.04)	0.088	.77
Age		40.29 (15.17)		40.35 (14.74)	0.002	.97
Household income		4.56 (2.66)		4.89 (2.49)	1.688	.20
Household size		2.30 (1.30)		2.38 (1.15)	0.341	.56

Note: *N* = sample size for the analysis (cases with missing values in one or more of the examined variables were not integrated); *M* = mean value; *SD* = standard deviation

Table A.4
Distribution of frequency of each choice-option per choice-set.

**Product**	**To consume vs. to offer**	**Options**	**1**	**2**	***„None“***	***„I don't know“***
Yogurt	consume	1 = optimal<br>2 = Exp1	3.0%	95.9%	0.9%	0.2%
		1 = optimal<br>2 = Exp2	16.7%	82.1%	0.5%	0.7%
		1 = Exp1<br>2 = Exp2	22.6%	75.6%	1.4%	0.4%
	offer	1 = optimal<br>2 = Exp1	36.4%	62.9%	0.4%	0.4%
		1 = optimal<br>2 = Exp2	61.1%	38.4%	0.2%	0.4%
		1 = Exp1<br>2 = Exp2	56.8%	35.3%	7.2%	0.7%
Cheese	consume	1 = optimal<br>2 = Exp1	2.5%	96.1%	0.7%	0.7%
		1 = optimal<br>2 = Exp2	14.2%	84.4%	0.5%	0.9%
		1 = Exp1<br>2 = Exp2	14.9%	82.4%	1.8%	0.9%
	offer	1 = optimal<br>2 = Exp1	23.1%	76.0%	0.4%	0.5%
		1 = optimal<br>2 = Exp2	50.2%	48.6%	0.2%	1.1%
		1 = Exp1<br>2 = Exp2	47.7%	48.4%	3.0%	0.9%
